# Supplementary material for: Content-rich biological network constructed by mining PubMed abstracts
Source: BMC Bioinformatics. 2004 Oct 8;5:147. doi: 10.1186/1471-2105-5-147 (PMC528731; doi:10.1186/1471-2105-5-147)
Supplement: Additional File 2 — The original results of the above study (non-essential files are deleted to keep the file size under the limit set by BMC bioinformatics). [file 1471-2105-5-147-S2.bz2 › chilibotAdditionalFile2/dip05/272ID1846781E1092/html/GSK3B_JUN.html]

 


 **GSK3B** and **JUN** 
  
Found 24 abstracts in PubMed, retrieved 05.  
 

 What does Google say? 
 PDF only 
| .edu only 

---

**Interactive relationship** (e.g. stimulation, inhibition, etc)

**Non-interactive relationship** (e.g. studied together, co-existance, homology, etc.)

- We have shown previously that glycogen synthase kinase 3 beta  [ **GSK3B** ] , cyclin dependent kinase 5, and c  **Jun**  NH terminal kinase become overactivated and hyperphosphorylate tau in heat shocked female rats.  Ref: 11805297 Proc Natl Acad Sci U S A, 2002
- We developed a sensitive ribonuclease RNase protection assay for the messenger RNA mRNA levels of immediate early IE genes c  **jun** , c fos and c myc, as well as platelet derived growth factor A  [ **GSK3B** ]  PDGF A, platelet derived growth factor beta receptor, transforming growth factor beta 1, and vascular endothelial growth factor.  Ref: 11888518 Atherosclerosis, 2002
